# Prevalence of lower extremity Peripheral artery disease among adult diabetes patients in Southwestern Uganda

**DOI:** 10.1186/1471-2261-14-75

**Published:** 2014-06-10

**Authors:** Samson Okello, Alexander Millard, Rogers Owori, Stephen Bambeiha Asiimwe, Mark Jacob Siedner, Joselyn Rwebembera, Laurence Anthony Wilson, Christopher Charles Moore, Brian Herb Annex

**Affiliations:** 1 Department of Medicine, Mbarara University of Science and Technology, P.O Box 1410, Mbarara, Uganda; 2 Department of Medicine, University of Virginia, Charlottesville, Virginia, USA; 3 Department of Medicine, Mbarara Regional Referral Hospital, Mbarara, Uganda; 4 Harvard Medical School, Boston, Massachusetts, United States of America; 5 The Robert M. Berne Cardiovascular Research Center, University of Virginia, Charlottesville, Virginia, USA

## Abstract

**Background:**

Peripheral artery disease (PAD) is a major complication of atherosclerosis. PAD can be diagnosed with low-cost diagnostic techniques and is associated with significant morbidity and mortality. While the major epidemiologic risk factors for PAD have been established in the western world, data from resource-poor countries are limited. We performed a cross-sectional study to determine the prevalence and correlates of PAD among patients with diabetes at Mbarara Referral Hospital in southwestern Uganda.

**Methods:**

We consecutively enrolled diabetes patients aged 50 years or greater presenting to the outpatient clinic. We collected blood for fasting lipid profile, HIV serology, and glycosylated hemoglobin, measured blood pressure and ankle brachial index, and administered the Edinburgh Claudication Questionnaire (ECQ). We also surveyed patients for other PAD risk factors. We used logistic regression to determine correlates of PAD.

**Results:**

We enrolled 229 diabetes patients. The median age of 60 years (IQR 55–66), and 146 (63.7%) were female. Fifty five patients (24%) had PAD (ABI of ≤ 0.9). Of these, 48 /55 (87.27%) had mild PAD (ABI 0.71-0.9) while 7/55 (12.73%) had moderate to severe PAD (ABI < 0.7). Amongst those with PAD, 24/55 (43.64%) reported claudication by the ECQ. Correlates of PAD included female sex (AOR 2.25, 95% CI 1.06 - 4.77, p = 0.034), current high blood pressure (AOR 2.59, 95% CI 1.25-5.33, p = 0.01), and being on a sulfonylurea–glibenclamide (AOR 3.47, 95% CI 1.55 - 7.76, p = 0.002).

**Conclusion:**

PAD was common in diabetic patients in southwestern Uganda. Given its low cost and ease of measurement, ABI deserves further assessment as a screening tool for both PAD and long term cardiovascular risk amongst diabetics in this region.

## Background

Cardiovascular disease is projected to become the leading cause of death in sub-Saharan Africa within the next two decades [1]. This epidemiologic shift will have a significant impact on health and relative burden of disease in the region [2]. Epidemiologic data on non-communicable diseases in this region are lacking and this is in part due to the non-availability and high cost of diagnostic tools.

Peripheral arterial disease (PAD) is among those non-communicable diseases that can be diagnosed without skilled technologists and at low cost by measurement of the ankle brachial index (ABI). The ABI, therefore, presents an attractive screening tool for PAD in resource-poor settings. Studies in resource-rich settings have shown that ABI is both sensitive and specific as a measure of lower extremity large vessel atherosclerosis [3-5]. In western countries, PAD patients are at high risk of myocardial infarction, stroke, amputations, and cardiovascular disease related death. Despite this, many patients with PAD are asymptomatic [6-8]. PAD, therefore, is an attractive target for screening because its early detection may not only help to delay its progression, but may also enable improved management of overall cardiovascular risk [9-14].

While in resource-rich settings, diabetes, smoking, and old age have been identified as the most important risk factors for PAD [15-17], in Sub-Saharan Africa, PAD is rarely diagnosed, in both diabetic and non-diabetic patients. Consequently, neither is the prevalence of PAD, nor its risk factors well understood in this region. A better understanding of PAD in a population with diabetes may help guide further research and identify potential intervention targets. For example, detection of PAD in such a high risk population may allow for the institution of relatively simple and low cost medical therapies with the potential to improve overall cardiovascular risk such as aspirin, 3-hydroxy-3-methyl-glutaryl-CoA reductase inhibitors (statins like artovastatin), and angiotensin converting enzyme (ACE) inhibitors [18]. We, therefore, performed a cross-sectional study to determine the prevalence and correlates of PAD among patients with diabetes at Mbarara Referral Hospital in Southwestern Uganda. We hypothesized that diabetic patients in sub-Saharan Africa would have a prevalence of PAD of 28% (with a 10% precision) based on what has been found in resource-rich settings (95% CI 19-29%) [19].

## Methods

### Study design and population

We consecutively enrolled all fasting diabetes patients of black race, aged ≥ 50 years [20], who presented to the diabetes outpatient clinic at Mbarara Regional Referral Hospital in Southwestern Uganda, between January and June 2012. To be included, patients had to have fasted on the morning of their clinic appointment. Routinely at this clinic, all patients are advised to fast on the morning of their clinic appointment to allow for the measurement of their fasting blood glucose. We excluded patients with lower limb amputations and those with suspicion of deep vein thrombosis (i.e., unilateral leg swelling, constant calf-muscle pain, and pain on foot flexion) for whom ABI testing would have been inappropriate. All patients gave written informed consent to participate and ethics approval was obtained from Mbarara University of Science and Technology and University of Virginia Institutional Review Boards. The study was registered with the Uganda National Council of Science and Technology.

### Data collection

#### ***Clinical and demographic variables***

We surveyed the patients to collect data on smoking history, hypertension, hyperlipidemia, stroke/transient ischemic attack, coronary artery disease, or chronic kidney disease. We also obtained data on the duration of diabetes and current medications. We categorized self-reported smoking as never, former, or current. A physician verbally administered the Edinburgh Claudication Questionnaire (ECQ). The ECQ has been shown in English speaking populations to be 91.3% sensitive and 99.3% specific for identifying claudication symptoms [21]. To adapt it to this population, the ECQ was pre-tested and translated into the local language (Runyankole). In addition, we ensured that it was administered by a physician fluent in the local language. Given that responses were quantitative, back translation was not done, and the data was entered directly into a database. A composite variable derived from the responses classified claudication as: none, atypical, or definite claudication. From the ECQ, definite claudication was defined as presence of calf pain, regardless of whether there was pain at other sites. Atypical claudication was defined as having pain in the thigh or buttock in the absence of calf pain. Finally, no claudication was defined as having pain in the hamstrings, feet, shins, joints, or any calf, thigh or buttock pain that appears to radiate, or no pain at all in any part of the leg [21].

### Measurement of ABI

For all patients, we measured the brachial blood pressure (BP) after a 5-minute rest in the supine position, using an aneroid sphygmomanometer (Welch Allyn® Tycos DS58 Hand Aneroids; Skaneateles Falls, New York, USA), recalibrated monthly according to the manufacturers’ manual. Two BP measurements were taken for each arm with a third reading performed only if the difference between the two was greater than 10 mmHg for systolic BP, or greater than 5 mmHg for diastolic BP. The mean of the closest two readings was then calculated and taken as the patient’s BP.

We measured ankle pressures using an aneroid sphygmomanometer while using a standardized Doppler ultrasonic device (8 MHz; Edan™ Sonotrax® MS3-14347A; Shekou, China) to accentuate the sounds over the posterior tibial and dorsalis pedis arteries. The ankle–brachial index for each leg was calculated by dividing the higher of two systolic BP readings at the ankle (i.e., one from the dorsalis pedis and the other from the posterior tibial artery) by the higher of the two brachial systolic BP readings from the ipsilateral arm (i.e., from the brachial artery). For all patients, measurements on both legs were obtained, and the lower value was considered as the patient’s ABI [20,22,23].

### Laboratory-based measurements

We measured fasting blood glucose level and glycosylated hemoglobin (HbA1c) levels using a hand-held glucometer (Abbott Optium Xceed® Diabetes Monitoring System; Abbott laboratories™, Illinois, USA) and a standard HbA1c rapid test kit (A1CNow + ®; Bayer™ HealthCare, Sunnyvale, CA, USA) respectively. We also tested patients for HIV serology using a standard algorithm and standard rapid testing kits, with a sensitive initial screen (Alere™ HIV-1/2 Determine® dipstick) followed by a more specific confirmatory test(STAT-PAK® HIV-1/2), with only the inconclusive results being subjected to a third rapid test as a tie breaker (Uni-Gold™ Recombigen® HIV). In addition, we obtained serum total cholesterol (TC), triglyceride (TG), high-density lipoprotein cholesterol (HDL-C), and low-density lipoprotein cholesterol (LDL-C) levels, using an automated clinical chemistry analyzer (Humastar® 180; Human Diagnostics Worldwide, Wiesbaden, Germany). The tests were performed at the Mbarara University Clinical Research Laboratory which participates in external quality assurance programs by the National Health Laboratory Service (Johannesburg, South Africa).

### Study definitions

We defined diabetes as a fasting glucose level ≥ 7.0 mmol/L (127 mg/dL), or taking medication for diabetes, or having been diagnosed with diabetes by a physician. We defined poor glycemic control as HbA1c > 7.0% (> 53 mmol/mol) [24]. Hypertension was defined as a systolic blood pressure ≥ 140 mmHg or diastolic blood pressure ≥ 90 mmHg or having been on hypertensive medications. Obesity was defined as a BMI ≥ 30 kg/m^2^, or a waist-to-hip ratio ≥ 0.81 or ≥ 0.96 for females and males respectively [25]. We defined hypercholesterolemia, high LDL-cholesterol, low HDL-cholesterol, hypertriglyceridemia, respectively, as serum cholesterol levels greater than 190 mg/L (4.9 mmol/L), LDL-cholesterol greater than 100 mg/dL (2.50 mmol/L), HDL-cholesterol levels less than 40 mg/L (1.03 mmol/L), and serum triglyceride levels greater than 200 mg/L (2.26 mmol/L) respectively [26]. We defined mild PAD as an ABI ≥ 0.71 but ≤ 0.90, moderate PAD as an ABI ≥ 0.40 but ≤ 0.70, and severe PAD as an ABI < 0.40 [20]. We considered a patient with PAD as being on PAD-appropriate medical therapy if they reported taking either aspirin or clopidigrel. However, for those with hypertension required an additional drug in form of a statin, or an ACEI to be considered as being on PAD appropriate therapy.

### Statistical methods

We summarized demographic and clinical characteristics, reporting proportions for categorical and binary variables, and median and interquartile range for continuous variables. We further divided the cohort into those with and without PAD and fit logistic regression models to determine the correlates PAD. In the multivariable models, we adjusted for sex, current high blood pressure, and taking glibenclamide). Statistical significance was set at *p* ≤ 0.05. We performed all statistical analyses using Stata 12 (StataCorp, College Station, Texas, USA).

## Results

Of 256 patients who were screened, 27 did not meet inclusion criteria and were excluded. Thirteen were aged ≤ 49 years, eight had no diabetes, one had an above-knee amputation, five declined participation, two withdrew consent, and one did not complete study procedures. Summary demographic and clinical characteristics for the remaining 229 patients are shown in Table 1. The cohort had a median age of 60 years, had been known to be diabetic for a median of 1.0 year (IQR 0.3-2.3), and had a median HbA1c of 8.1% (6.7-10.1). Twenty-nine percent were taking insulin, and 41% were former or current smokers.

**Table 1:** Characteristics of the study population

**Characteristic**	**N = 229**
Age (years), median (IQR)	60 (55–66)
Female sex, n (%)	146 (63.7)
Current smoker, n (%)	12 (5.2)
Former smoker, n (%)	82 (35.8)
Never smoked	135 (59)
Duration of diabetes (years), median (IQR)	1.0 (0.3–2.3)
Family history of diabetes, n (%)	78 (34.1)
No Claudication, n (%)	126 (55.0)
Atypical Claudication, n (%)	54 (23.6)
Definite Claudication, n (%)	49 (21.4)
Self-reported hypertension, n (%)	113 (49.3)
HIV infection, n (%)	18 (7.9)
Diabetes treatment^π^, n (%)	
Metformin only	112 (51.6)
Glibenclamide only	2 (0.9)
Glibenclamide and Metformin	34 (15.7)
Insulin only	52 (24)
Insulin and Metformin	15 (6.9)
Aspirin, n (%)	17 (7.4)
BMI (kg/m^2^), median (IQR)	26.3 (22.6–29.4)
Waist-to-hip ratio, median (IQR)	1 (1–1)
Systolic blood pressure (mmHg), median (IQR)	140 (124–160)
Diastolic blood pressure (mmHg), median (IQR)	80 (70–90)
Fasting blood sugar (mmol/L), median (IQR)	8.6 (6.6–13.7)
HbA1c (%), median (IQR)	8.1 (6.7–10.1)
HDL^£^-Cholesterol (mg/dL), median (IQR)	48 (39–58)
LDL^¥^-Cholesterol (mg/dL), median (IQR)	81 (63–96)
Triglycerides (mg/dL), median (IQR)	116 (83–179)
Total cholesterol (mg/dL), median (IQR)	268 (212–307)

IQR: Inter quartile range; HIV: Human immunodeficiency virus; BMI: body mass index; HDL^£^: High-density Lipoprotein- cholesterol; LDL^¥^: Low-density lipoprotein-cholesterol; HbA1c: glycated hemoglobin.

Diabetes treatment^π^: A total of 217 patients were on diabetes medication.

The prevalence of PAD (ABI of ≤ 0.9) was 24% (55/229). Among the patients with PAD, 48/55 (87%) had mild PAD (ABI 0.71-0.90) while 7/55 (13%) had moderate to severe PAD (ABI < 0.70). Based on the ECQ, 31/55 (56%) of those with PAD were asymptomatic, whereas only 11/55 (20%) showed definite claudication, and 13/55 (24%) had atypical claudication symptoms. Additionally, among those with PAD, 6/55 (11%) were taking aspirin, and only 1 patient was taking a statin. Thirty three of 113 (29.2%) patients who had hypertension with concurrent PAD were on an ACE inhibitor or ARB.

In the unadjusted analysis (Table 2), female sex, self-reported hypertension, current high blood pressure, and duration of diabetes were associated with PAD. In the multivariable adjusted analysis, (Table 3), female sex (AOR 2.25, 95% CI 1.06–4.77, p = 0.034), current high blood pressure (AOR 2.59, 95% CI 1.25–5.33, p = 0.01), and being on sulfonylurea–glibenclamide (AOR 3.47, 95% CI 1.55–7.76, p = 0.002) were independently associated with PAD.

**Table 2:** Unadjusted analysis of predictors of Peripheral Artery Disease

**Characteristic**	**OR (95% CI)**	**p-value**
Female sex	2.47 (1.22–5.01)	0.0001
Smoking history	1.04 (0.56–1.93)	0.894
Duration of diabetes > 5 years	3.38 (0.94–12.15)	0.062
Family history of diabetes	1.37 (0.73–2.56)	0.326
Presence of claudication symptoms	0.93 (0.51–1.71)	0.819
Self-reported hypertension^¥^	1.96 (0.95–3.26)	0.072
Current elevated blood pressure^β^	2.73 (1.42–5.25)	0.003
HIV infection	0.61 (0.17–2.19)	0.451
Metformin use	0.74 (0.39–1.42)	0.367
Glibenclamide use	3.16 (1.50–6.66)	0.002
Insulin use	1.24 (0.65–2.38)	0.517
Obesity, BMI ≥ 30 Kg/m^2^	1.23 (0.59–2.53)	0.576
Waist-to-hip ratio ≥ 0.81 (F) or ≥ 0.96 (M)	0.83 (0.21–3.26)	0.796
Cholesterol ≥ 190 mg/dL	0.9 (0.41–1.98)	0.794
HDL^€^ ≤ 40 mg/dL	1.21 (0.61–2.41)	0.581
LDL^α^ ≥ 100 mg/dL	1.39 (0.69–2.78)	0.355
TG^∞^ ≥ 200 mg/dL	0.75 (0.34–1.67)	0.483
FBS^μ^ < 3.9 or > 7.2 mmol/L	0.87 (0.46–1.65)	0.679
HbA1c^£^ > 7.0% (53 mmol/mol)	0.75 (0.39 - 1.41)	0.368

Self-reported hypertension^¥^: prior diagnosis of hypertension and taking antihypertensive drugs; Current elevated blood pressure^β^: clinic systolic blood pressure ≥ 140 mmHg and/or diastolic blood pressure ≥ 90 mmHg; OR: Odds ratio; BMI: body mass index; HDL^€^: High-density Lipoprotein- cholesterol; LDL^α^: Low-density lipoprotein-cholesterol; TG^∞^: Triglycerides; FBS^μ^: fasting blood sugar; HbA1c^£^: glycated hemoglobin.

**Table 3:** Multivariable adjusted predictors of Peripheral Artery Disease

**Characteristic**	**AOR**	**95% CI**	**p-value**
Glibenclamide use	3.47	1.55 - 7.76	0.002
Current high blood pressure^β^	2.59	1.25 - 5.33	0.01
Female sex	2.25	1.06 - 4.77	0.034

Current high blood pressure ^β^: clinic measured systolic blood pressure ≥ 140 mmHg and/or diastolic blood pressure ≥ 90 mmHg; AOR: Adjusted odds ratio.

## Discussion

To the best of our knowledge, this is the first report of the prevalence of PAD in patients with diabetes in Sub-Sahara Africa. The prevalence of PAD of 24%, as found in our study based on ABI, was substantially higher than previous estimates (0.2–3.4%) which were based on clinical parameters such as weak or absent peripheral pulses, or development of dry gangrene [27,28]. However, our finding is comparable to the upper end of the range (1.7–28%) found when ABI was measured by echographic and doppler flow velocity waveform analysis methods [2]. The prevalence of PAD in our study population is also well within the range of several screening studies in the US that used a similar screening technique, and, in African American diabetic patients, found a prevalence of PAD between 14-30% [29-31]. The low proportion of smokers in our study (5.2%) reflects the low national smoking prevalence in Uganda [32].

In the multivariable-adjusted analysis, we found that female sex, current high blood pressure, and use of glibenclamide were predictive of PAD. Population based studies have previously reported an association of PAD with female sex [33] and hypertension [34], which we have also shown in our study. In our clinic, the majority of the patients are of the female sex [35]. This pattern may be because females generally outnumber males in the Ugandan population and, in addition, they tend to have better health seeking behaviour [36,37]. We were unable to assess independent association of smoking with PAD because of a very low prevalence of smoking. A low prevalence of smoking has been reported in the general Ugandan population, and the prevalence is even lower in females [32,35,37]. It is therefore possible that the observed lack of association with smoking with PAD may be due to the high female proportion in the setting of a low prevalence of smoking in females. Given this observation, the finding that female sex was an independent predictor of PAD may or may not represent a real association in this population, and, therefore, deserves further investigation in future studies.

Despite international recommendations for use of aspirin, ACEIs, and statins for PAD [18], our study shows that diabetes patients are undertreated with regard to atherosclerotic risk factor modification. This may be because patients with diabetes can be asymptomatic even in the presence of serious cardiovascular complications. PAD screening in these patients may therefore enhance the awareness of active cardiovascular complications and allow for more risk factor treatment. We do appreciate that there can be a high rate of noncompliance to medication for chronic non-communicable disease is in our setting. However, our finding of a high prevalence of PAD in the context of under treatment, may suggest the existence of prevention opportunities in this population.

Although sulfonylureas, including glyburide and gliclazide, have been associated with an increased mortality from acute coronary syndrome in type 2 diabetes patients [38], we know of no prior study that has reported an association between glibenclamide use and PAD. This may, therefore, be a spurious finding from our study. However, the association was independent of concurrent metformin use, diabetes duration and severity (as assessed through HbA1c). In addition, those taking insulin were not at increased risk of PAD, indicating the increased risk of PAD in those on glibenclamide may not be related to the known risk of hypoglycemia that occurs with glibenclamide. Thus, our findings may suggest an independent association between PAD and glibenclamide use (Table 3) requiring further investigation.

Previous studies have reported that even among patients with a low ABI and thus confirmed PAD, absence of “leg” symptoms is not very useful in excluding PAD [6,20]. We found that only 20% of those with PAD had definite claudication, and that over half had no claudication symptoms on the ECQ. This is similar to what has been found by studies in resource rich settings where this tool showed 20% sensitivity in diagnosing PAD [16]. Our data similarly suggest that claudication symptoms are not very helpful in excluding PAD in Sub-Saharan Africa.

The findings in this report are subject to a number of limitations. First, this was a cross sectional study in a population with diabetes where the risk for PAD is high. Second, the patients studied were from a referral center. These observations imply that our findings may not necessarily represent the general Ugandan population, or those patients presenting in primary care settings. In addition, our findings may not reflect the prevalence of PAD if it was measured using other screening criteria. However, our data suggest that criteria applied in resource-rich settings may as well be applicable in the resource-poor setting.

## Conclusion

In conclusion, PAD was common among diabetes outpatients ≥ 50 years old presenting at a referral hospital in southwestern Uganda. Given that this is a group at high risk of cardiovascular complications, the high prevalence of PAD in a setting of under-treatment of general cardiovascular risk, call for further study of PAD in this population. For example, future studies could investigate whether screening for PAD in this population using ABIs might lead to increased use of evidence-based treatments that are known to reduce general cardiovascular risk. Future research should also attempt to determine the prevalence and risk factors of PAD in more generalized populations in sub-Saharan Africa.

## Abbreviations

ABI: Ankle-Brachial Index, PAD, Peripheral Artery Disease, AOR, Adjusted Odds Ratio.

## Competing interests

The authors prepared this report in their roles as employees of their respective institutions. They have no financial or other potential conflict of interest with regard to this manuscript.

## Author contributions

SO, AM, RO, SBA, and BHA conceived the study, collected the data, contributed to discussion, and reviewed, edited, and wrote the manuscript. MJS, JR, LAW, CCM, and BHA contributed to discussion and reviewed and edited the manuscript. S.O and C.C.M analyzed the data. SO is the guarantor of this work and, as such, had full access to all the data in the study and takes responsibility for the integrity of the data and the accuracy of the data analysis. All authors read and approved the final manuscript.

## Pre-publication history

The pre-publication history for this paper can be accessed here:

http://www.biomedcentral.com/1471-2261/14/75/prepub
